# *Arabidopsis thaliana* Ei-5: Minor Vein Architecture Adjustment Compensates for Low Vein Density in Support of Photosynthesis

**DOI:** 10.3389/fpls.2018.00693

**Published:** 2018-06-01

**Authors:** Jared J. Stewart, Stephanie K. Polutchko, Barbara Demmig-Adams, William W. Adams

**Affiliations:** Department of Ecology and Evolutionary Biology, University of Colorado Boulder, Boulder, CO, United States

**Keywords:** *Arabidopsis thaliana*, phloem, photosynthesis, transpiration, vein density, xylem

## Abstract

An *Arabidopsis thaliana* accession with naturally low vein density, Eifel-5 (Ei-5), was compared to Columbia-0 (Col-0) with respect to rosette growth, foliar vein architecture, photosynthesis, and transpiration. In addition to having to a lower vein density, Ei-5 grew more slowly, with significantly lower rates of rosette expansion, but had similar capacities for photosynthetic oxygen evolution on a leaf area basis compared to Col-0. The individual foliar minor veins were larger in Ei-5, with a greater number of vascular cells per vein, compared to Col-0. This compensation for low vein density resulted in similar values for the product of vein density × phloem cell number per minor vein in Ei-5 and Col-0, which suggests a similar capacity for foliar sugar export to support similar photosynthetic capacities per unit leaf area. In contrast, the product of vein density × xylem cell number per minor vein was significantly greater in Ei-5 compared to Col-0, and was associated not only with a higher ratio of water-transporting tracheary elements versus sugar-transporting sieve elements but also significantly higher foliar transpiration rates per leaf area in Ei-5. In contrast, previous studies in other systems had reported higher ratios of tracheary to sieve elements and higher transpiration rate to be associated with higher – rather than lower – vein densities. The Ei-5 accession thus further underscores the plasticity of the foliar vasculature by illustrating an example where a higher ratio of tracheary to sieve elements is associated with a lower vein density. Establishment of the Ei-5 accession, with a low vein density but an apparent overcapacity for water flux through the foliar xylem network, may have been facilitated by a higher level of precipitation in its habitat of origin compared to that of the Col-0 accession.

## Introduction

The maximal capacity of a leaf to carry out photosynthesis is closely coordinated with the capacity of the foliar vasculature for sugar and water transport, and these features co-vary in response to the growth environment as has been documented in several genotypes of the winter annual *Arabidopsis thaliana* (Cohu et al., 2013a,b; Adams et al., 2016; Stewart et al., 2016, 2017a,b, 2018). Foliar vascular modulation in response to the growth environment includes adjustment in vein density (Amiard et al., 2005; Adams et al., 2007, 2013a, 2016; Dumlao et al., 2012; Muller et al., 2014b; Stewart et al., 2016, 2017a,b), number of vascular cells per minor vein (Adams et al., 2013a, 2016; Cohu et al., 2013a,b, 2014; Muller et al., 2014b; Stewart et al., 2016, 2017a,b), size of individual vascular cells (Adams et al., 2013a; Muller et al., 2014b; Stewart et al., 2017a,b), and the ultrastructure of phloem transfer cells (Amiard et al., 2005, 2007; Adams et al., 2014a, 2016). Such modulation of foliar vascular features is predicted to support photosynthesis as the need for water import and sugar export change in response to the growth environment. Leaves need to replace water lost during CO_2_ uptake in order to keep stomata open for photosynthesis (Brodribb et al., 2007; Hölttä et al., 2017; Sperry et al., 2017). In addition, expedient removal of sugar produced in photosynthesis counteracts carbohydrate build-up in leaves and downregulation of photosynthesis (Krapp et al., 1993; Krapp and Stitt, 1995; Pego et al., 2000; Kasai, 2008; Adams et al., 2013b, 2014b).

The foliar vascular architecture is highly flexible and exhibits acclimatory adjustment of its components in response to environmental conditions and dependent on genotype. However, minor vein anatomical features consistently correlated with leaf function across this variation among genotypes and environmental conditions. Under all conditions, adjustment of minor-vein phloem features involved in sugar-loading and sugar-export capacity closely matched adjustment of photosynthetic capacity (Adams et al., 2013a, 2014a, 2016; Cohu et al., 2013a,b, 2014; Muller et al., 2014b; Stewart et al., 2016, 2017a,b). Conversely, adjustment of vein density and xylem features of minor veins involved in leaf hydraulic conductivity closely matched transpiration rates under all conditions (Adams et al., 2016; Stewart et al., 2016, 2017a,b). For instance, acclimation to high versus low light involved parallel upregulation of foliar vascular features associated with higher capacities for both sugar and water transport, of photosynthetic capacities, and of transpiration rates (Stewart et al., 2017a,b).

In addition to concomitant changes in foliar vascular features and photosynthetic capacity, the foliar vasculature can also be packaged differently to support similar photosynthetic capacities. For instance, leaves of summer annuals typically exhibit numerous minor veins that are of small size, whereas winter annuals typically have leaves with fewer, but larger minor veins (Demmig-Adams et al., 2014; Polutchko et al., 2018; see also Cohu et al., 2014; Stewart et al., 2016). Under the growth conditions and in the *A.* *thaliana* genotypes examined thus far, a higher vein density was consistently associated with a higher proportion of transpiration to photosynthesis, xylem to phloem, and water conduits to sugar conduits (Adams et al., 2016; Stewart et al., 2016, 2017a,b, 2018). Investigations of mutants with altered foliar vascular phenotypes have also reported corresponding differences in leaf functioning relative to wild-type (e.g., Caringella et al., 2015; Feldman et al., 2017). The present study uses a naturally occurring low-vein-density phenotype to evaluate whether these associations are fixed or exhibit acclimatory flexibility.

Some *A. thaliana* accessions exhibit naturally occurring unusual foliar vein densities as rare occurrences in nature. In a survey of over 250 *A. thaliana* accessions, Candela et al. (1999) discovered only two (Eifel-5 and Blackmount-1) with foliar vein densities that were “unequivocally different” from others in the study (see also Pérez-Pérez et al., 2009). Of these two accessions, Eifel-5 (Ei-5) had the most simplified venation pattern, resulting from a recessive allele of *HVE*/*CAND1* (Alonso-Peral et al., 2006) – a gene involved in auxin signaling (see also Cheng et al., 2004). We asked whether such a genetically imposed constraint limits the ability of this accession to deliver water and export sugars, thereby resulting in lower rates of photosynthesis. Or does minor-vein composition and architecture of the Ei-5 accession undergo compensatory adjustments to maintain water- and sugar-transport capacities per unit leaf area? The present study compared foliar anatomical and physiological characteristics of the low-vein-density Ei-5 accession with the widely studied Col-0 accession of *A. thaliana*.

## Materials and Methods

### Plant Material

**FIGURE 1:**
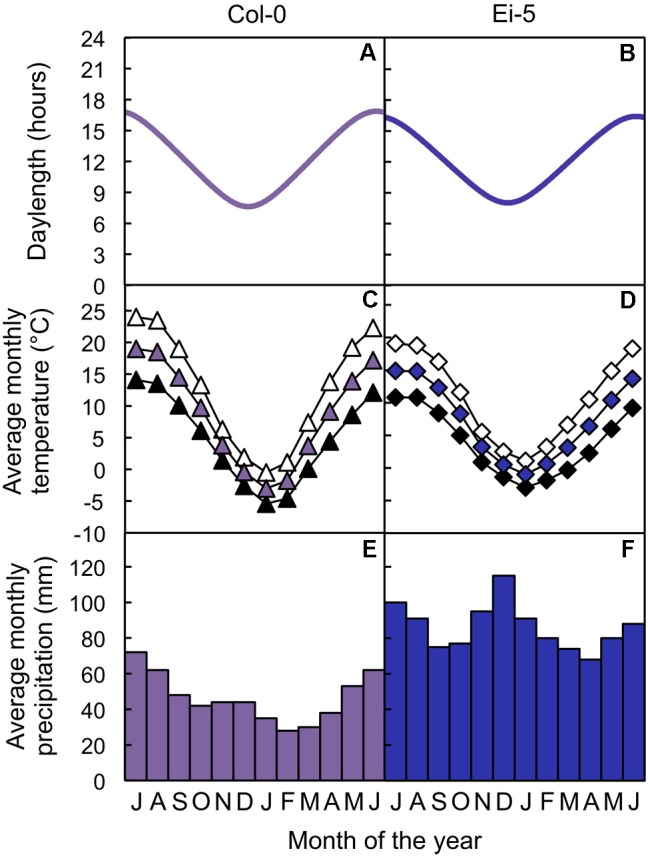
**(A,B)** Photoperiods, **(C,D)** average monthly maximum (open symbols), mean (colored symbols), and minimum (closed symbols) temperatures, and **(E,F)** average monthly precipitation totals of the sites from which the **(A,C,E)** Col-0 (violet) and **(B,D,F)** low-vein-density Ei-5 (indigo) accessions of *Arabidopsis thaliana* originate.

Seeds of both the Columbia-0 (Col-0) and low-vein-density Eifel-5 (Ei-5; CS1128) accessions of *A. thaliana* were obtained from The Arabidopsis Information Resource^1^. The Columbia accession originates from northwestern Poland (52.7°N, 15.7°E; from El-Lithy et al., 2004; see also Rédei, 1992; Koornneef and Meinke, 2010) and the Eifel accession originates from western Germany (50.3°N, 6.3°E; from the Master Accession List on 1001 Genomes’ website^2^), the sites of which are separated by 702 km (calculated with Latitude/Longitude Distance Calculator on NOAA’s website^3^). Daylength data of the geographic sites from which the accessions originate were obtained from The United States Naval Observatory’s website^4^. Altitude, temperature, and precipitation data of the geographic sites from which the accessions originate were obtained from the WorldClim database (Hijmans et al., 2005^5^) with DIVA-GIS software (Hijmans et al., 2012^6^). The Col-0 and low-vein-density Ei-5 accessions of *A. thaliana* thus experienced similar photoperiods (**Figures 1A,B**) and similar temperatures (**Figures 1C,D**) over the course of the year, but the Ei-5 accession received almost twice as much annual precipitation compared to Col-0 (1034 mm vs. 558 mm, respectively; **Figures 1E,F**).

### Growth Conditions

Col-0 and Ei-5 were grown under contrasting light intensities of 100 μmol vs. 1000 μmol photons m^-2^ s^-1^ at a common leaf temperature of 20°C as previously described (Stewart et al., 2017a). Seeds were incubated in water at 4°C for 4 days and germinated in six-pack seed starting trays (maximal volume of 50 mL) containing soil (Fafard Growing Mix 2; Sun Gro Horticulture, Agawam, MA, United States) in Conviron E15 and PGR15 growth chambers (Controlled Environments Ltd., Winnipeg, MB, Canada) with air temperatures set at 25°C during a 9-h photoperiod of 100 or 1000 μmol photons m^-2^ s^-1^ and 20°C during the 15-h dark period. Following germination and the removal of excess seedlings, individual seedlings were transplanted with soil from their respective cells into larger pots (maximal volume of 2.9 L). Under the high light regime, seedlings were transferred to a constant air temperature of 15°C for 1 week before being transferred to the final growth temperature of 12°C, which resulted in leaf temperatures of 20°C during the photoperiod and 12°C during the dark period. Under the low light conditions, seedlings were transferred to 20°C/15°C (light/dark) for 1 week before being transferred to the final growth temperature of 20°C during the photoperiod and 12°C during the dark period. Plants received nutrients (Jack’s Professional LX Water-Soluble Fertilizer, J.R. Peters, Inc., Allentown, PA, United States) via a Dosatron D14MZ2 injector system (Dosatron International, Inc., Clearwater, FL, United States) every other day and water on days when they did not receive nutrients (Stewart et al., 2017b).

### Photosynthesis and Transpiration

Photosynthetic capacity was determined as the light- (1500 [low-light plants] or 2000 [high-light plants] μmol photons m^-2^ s^-1^) and CO_2_- (50,000 ppm) saturated rate of oxygen evolution using actinic light sources and leaf-disk oxygen electrode chambers (Hansatech Instruments, King’s Lynn, Norfolk, United Kingdom; Delieu and Walker, 1981; Adams et al., 2002) coupled to circulating water baths (Fisher Scientific, Pittsburgh, PA, United States) set to 25°C. For measurements of transpirational water loss and CO_2_ uptake, plants were removed from the growth chamber one at a time approximately 5 h into the photoperiod and exposed to either 100 or 1000 μmol photons m^-2^ s^-1^ (the light intensity during the photoperiod for plants grown under low or high light intensity, respectively) in air (409 ± 30 ppm CO_2_) for approximately 5 min, resulting in leaf temperatures of 27.4 ± 1.4°C and vapor-pressure deficits of 2.14 ± 0.23 kPa (*n* = 16). Transpiration rate and CO_2_ uptake were measured with an LCi Portable Photosynthesis System (ADC Bioscientific, Hoddesdon, England, United Kingdom).

### Leaf and Vein Anatomical Metrics

Rosette area was determined as the total light-exposed leaf area per plant from images taken directly above the plants and quantified using ImageJ software (Rasband WS, National Institutes of Health, Bethesda, MD, United States) as described in Stewart et al. (2015). Leaf mass per area was determined from leaf disks that were dried at 70°C for 1 week. Foliar vein density was assessed as vein length per leaf area from leaf sections that had been chemically cleared by soaking leaf tissue in 70% (v/v) ethanol and then 5% (w/v) NaOH (described in Stewart et al., 2017a). For the determination of foliar vein orders, chemically cleared leaf sections were stained with a dilute (0.01% [w/v]) Safranin O solution and imaged with a high-resolution scanner (Perfection 3200 Photo; Epson America, Inc., Long Beach, CA, United States). Foliar vein cross-sectional area and cell numbers were determined as described in Cohu et al. (2013a) from semi-thin (0.7–0.9 μm) cross-sections that were obtained with an Ultracut E microtome (Reichert Technologies Life Science, Buffalo, NY, United States) from leaf pieces (approximately 2 mm × 2 mm) that had been fixed in glutaraldehyde and paraformaldehyde, dehydrated through an acetone series, and embedded in Spurr resin (Spurr, 1969) as described by Dumlao et al. (2012; see also Stewart et al., 2017b). For Ei-5 plants grown under low light, five to six foliar veins per plant were characterized — rather than the seven to ten per plant described in Cohu et al. (2013a) — due to the relative scarcity of appropriate foliar vein cross-sections. Foliar vein density, cross-sectional area, and cell numbers were measured with ImageJ from images that were obtained using an Axioskop 20 light microscope (Carl Zeiss AG, Oberkochen, Germany) and an OptixCam Summit Series digital microscope camera and OCView software (The Microscope Store, LLC, Roanoke, VA, United States).

### Statistical Analyses

Differences between Col-0 and the low-vein-density Ei-5 accession were assessed via *t*-tests. The effects of genotype, growth light intensity, and genotypic response to growth light intensity were assessed via two-way analysis of variance. The relationship between groups with consideration of multiple variables was assessed via principal component analysis (see Polutchko et al., 2018). Non-linear growth curves for rosette area data were generated using a three-parameter logistic model (Paine et al., 2012), which has been used for similar analyses of *A. thaliana* (e.g., Tessmer et al., 2013). All statistical analyses were conducted with JMP software (Pro 13.1; SAS Institute, Inc., Cary, NC, United States).

## Results

**FIGURE 2:**
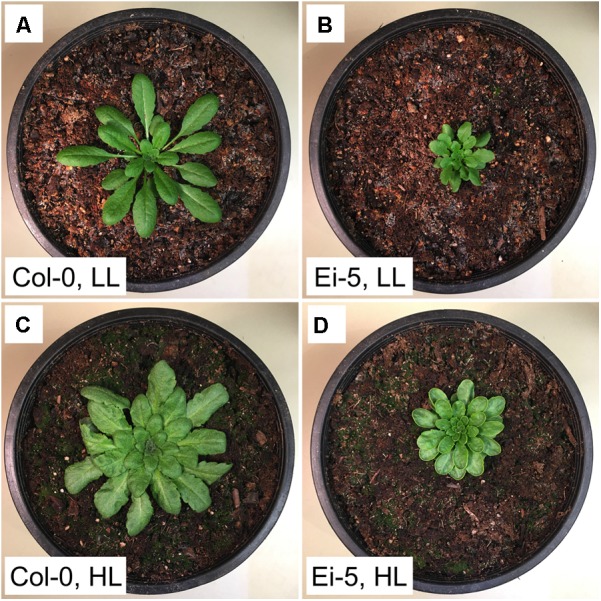
Photographic images of the **(A,C)** Col-0 and **(B,D)** low-vein-density Ei-5 accessions of *A. thaliana* grown under a 9-h photoperiod of **(A,B)** 100 (LL) or **(C,D)** 1000 (HL) μmol photons m^-2^ s^-1^ at a leaf temperature of 20°C for 42 days.

**FIGURE 3:**
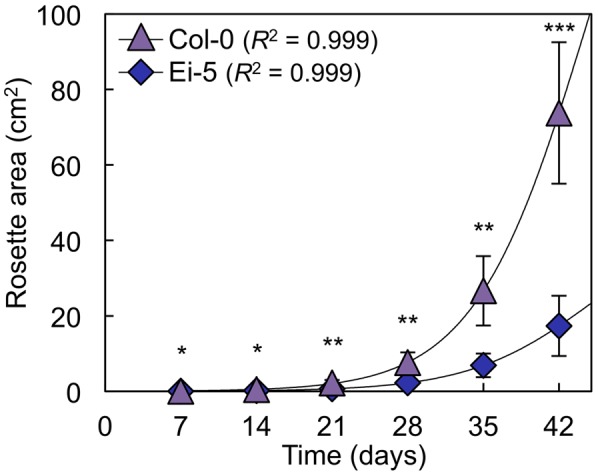
Total light-exposed rosette area per plant determined at weekly intervals for the Col-0 (violet triangles) and low-vein-density Ei-5 (indigo diamonds) accessions of *A. thaliana* during 42 days of growth under a 9-h photoperiod of 1000 μmol photons m^-2^ s^-1^ at a leaf temperature of 20°C. Non-linear growth curves were generated using three-parameter logistic models (Paine et al., 2012). Mean values ± standard deviations, *n* = 4. The asterisks denote significant differences between accessions in rosette size; ^∗^*P* < 0.05, ^∗∗^*P* < 0.01, and ^∗∗∗^*P* < 0.001.

**FIGURE 4:**
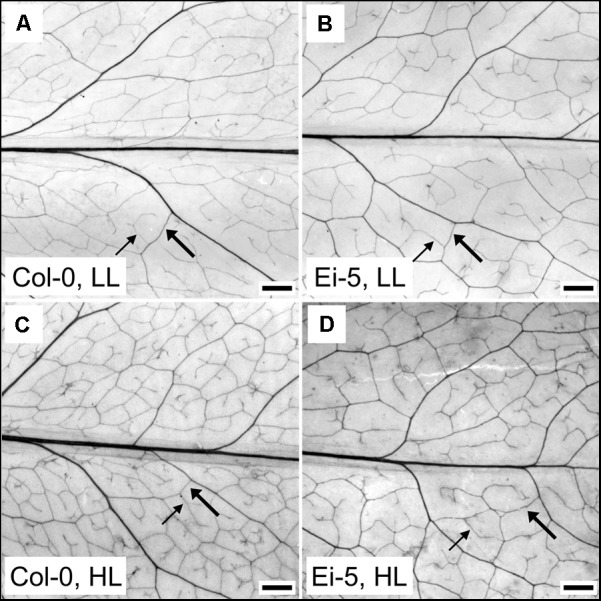
Images of representative chemically cleared leaves of the **(A,C)** Col-0 and **(B,D)** low-vein-density Ei-5 accessions of *A. thaliana* grown under a 9-h photoperiod of **(A,B)** 100 (LL) or **(C,D)** 1000 (HL) μmol photons m^-2^ s^-1^ at a leaf temperature of 20°C. To better visualize the leaf vascular network, the contrast of each image was enhanced using the “Auto Contrast” feature in Adobe Photoshop CS4 (Adobe Photosystems, Inc., San Jose, CA, United States). Representative third (3°) and fourth (4°) order minor veins are indicated with thick and thin arrows, respectively. Scale bar = 1 mm.

**FIGURE 5:**
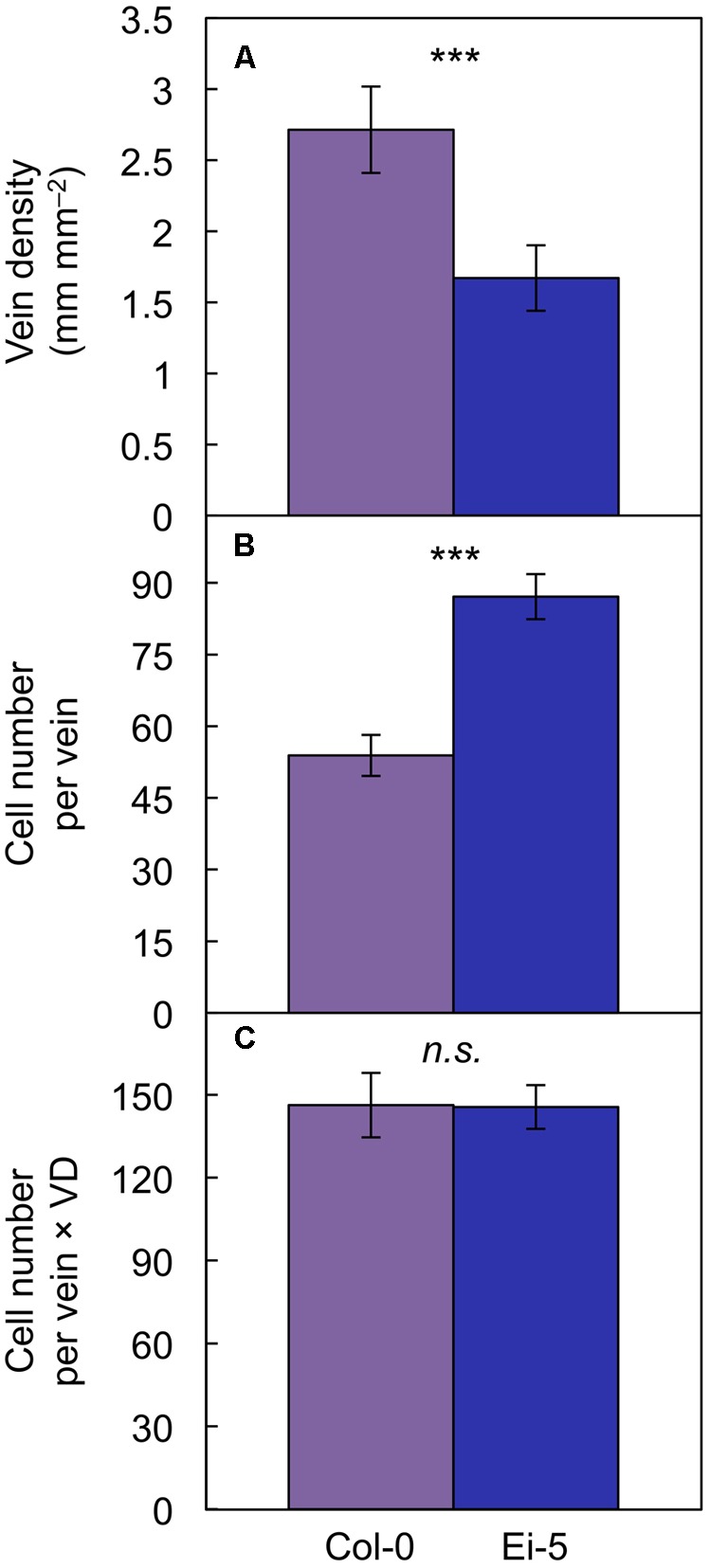
The **(A)** foliar vein density, **(B)** number of cells per minor vein, and **(C)** product of vein density and the number of cells per minor vein in the Col-0 (violet columns) and low-vein-density Ei-5 (indigo columns) accessions of *A. thaliana* grown under a 9-h photoperiod of 1000 μmol photons m^-2^ s^-1^ at a leaf temperature of 20°C. Mean values ± standard deviation for **(A)** and ±standard error for **(B,C)**; *n* = 4. The asterisks denote significant differences between the mean values; ^∗∗∗^*P* < 0.001 and *n.s*., not significantly different.

**FIGURE 6:**
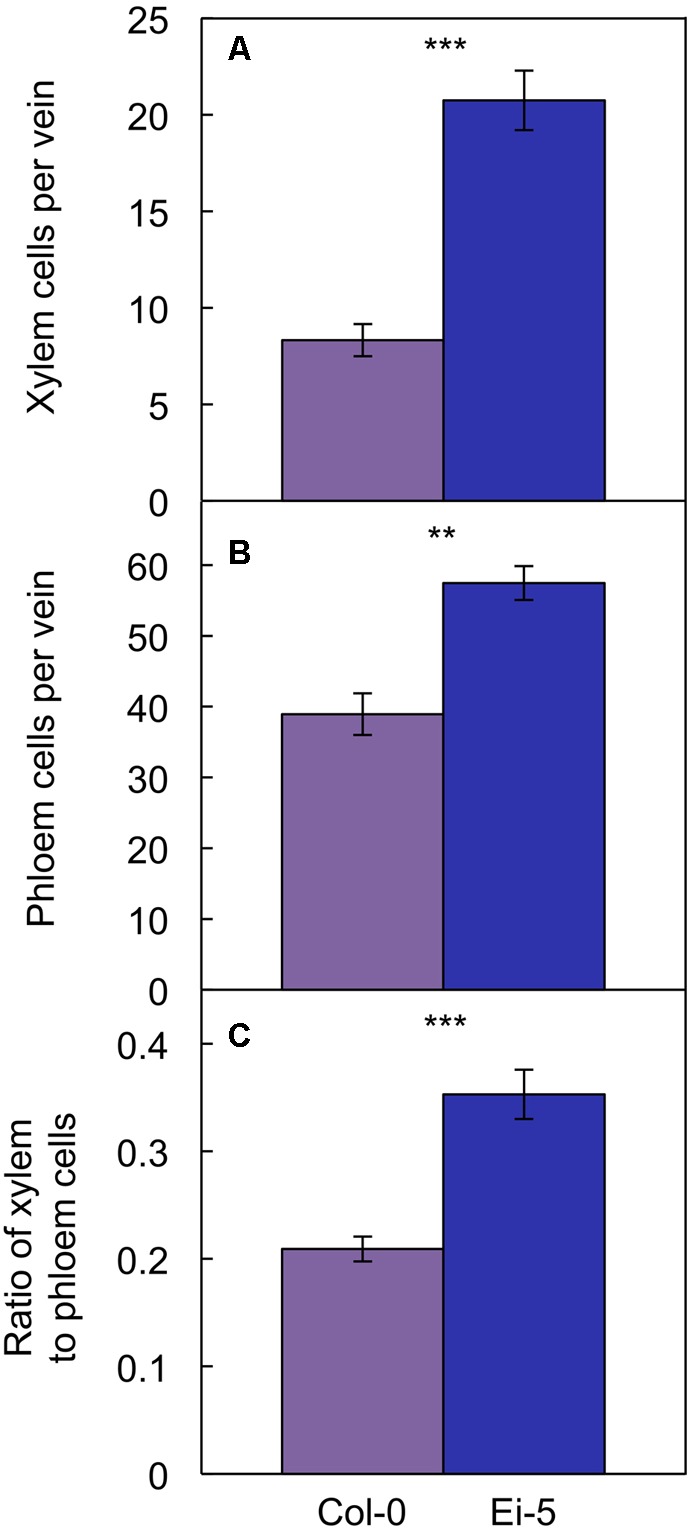
The number of **(A)** xylem cells per minor vein, **(B)** phloem cells per minor vein, and **(C)** ratio of xylem to phloem cells in minor veins of the Col-0 (violet columns) and low-vein-density Ei-5 (indigo columns) accessions of *A. thaliana* grown under a 9-h photoperiod of 1000 μmol photons m^-2^ s^-1^ at a leaf temperature of 20°C. Mean values ± standard errors; *n* = 4. The asterisks denote significant differences between the mean values; ^∗∗^*P* < 0.01, ^∗∗∗^*P* < 0.001.

The rosettes of the low-vein-density Ei-5 accession were consistently much smaller than those of Col-0 when grown under either low or high light at 20°C (**Figure 2**). The difference in rosette size was significant after a short period of growth, and became increasingly more pronounced over time as plants continued to grow (**Figure 3**). All vein orders (from largest [i.e., 1°] to smallest [i.e., 4°] veins) were present in the leaves of the two accessions under both low and high growth light (**Figure 4**). Minor-vein density was significantly lower in Ei-5 compared to Col-0 (**Figure 5A** and **Table 1**), but the number of vascular cells per minor vein was significantly greater in Ei-5 (**Figure 5B** and **Table 1**). These latter two differences compensated for each other, and total vascular cell number normalized for vein density was the same in the two accessions, as ascertained by multiplication of foliar vein density × the number of vascular cells per minor vein (**Figure 5C** and **Table 1**). Additional complexity was revealed when minor-vein xylem and phloem components were considered separately. The difference in cell number per minor vein between the two accessions grown under high light was even more pronounced for xylem (**Figure 6A**) than for phloem (**Figure 6B**), resulting in a greater ratio of xylem to phloem cells in the minor veins of Ei-5 compared to Col-0 (**Figure 6C** and **Table 1**).

**Table 1:** Leaf minor vein features and gas exchange for the Col-0 and low-vein-density Ei-5 accessions of *Arabidopsis thaliana* grown under a 9-h photoperiod of 100 μmol photons m^-2^ s^-1^ at a leaf temperature of 20°C.

	Low light		
Parameter	Col-0	Ei-5	*P*
Minor vein density (mm mm^-2^)	2.12 @ 0.10	1.52 @ 0.12	^∗∗∗^
Cells per minor vein	39.4 @ 1.9	54.9 @ 1.4	^∗∗∗^
Cells per minor vein × vein density	83.8 @ 4.1	83.6 @ 2.1	*n.s.*
Xylem cells per minor vein	5.8 @ 0.6	10.7 @ 0.9	^∗∗^
Phloem cells per minor vein	30.6 @ 1.2	39.7 @ 1.7	^∗∗^
Ratio of xylem to phloem cells	0.187 @ 0.017	0.273 @ 0.034	^∗^
Tracheary elements per minor vein	3.46 @ 0.37	6.53 @ 0.47	^∗∗^
Sieve elements per minor vein	7.0 @ 0.4	10.2 @ 0.6	^∗∗^
CCs + PCs per minor vein	23.7 @ 0.8	29.5 @ 1.2	^∗∗^
Ratio of tracheary to sieve elements	0.509 @ 0.054	0.666 @ 0.069	*n.s.*
Photosynthetic capacity (μmol O_2_ m^-2^ s^-1^)	16.0 @ 3.3	16.0 @ 0.8	*n.s.*
Phloem cells per minor vein × vein density	65.1 @ 2.5	60.4 @ 2.6	*n.s.*
Sieve elements per minor vein × vein density	14.8 @ 0.9	15.5 @ 0.9	*n.s.*
CCs + PCs per minor vein × vein density	50.3 @ 1.8	44.9 @ 1.9	^∗^
Transpiration ratio (mol H_2_O mol^-1^ CO_2_)	321 @ 59	705 @ 206	^∗∗^
Xylem cells per minor vein × vein density	12.4 @ 1.4	16.3 @ 1.3	^∗^
Tracheary elements per minor vein × vein density	7.36 @ 0.79	9.95 @ 0.71	^∗^

*Mean values ± standard deviation for vein density and gas exchange parameters and ± standard error for minor vein parameters; n = 3–4. The asterisks denote significant differences between the mean values; ^∗^P < 0.05, ^∗∗^P < 0.01, ^∗∗∗^P < 0.001, and n.s., not significantly different. CC, companion cells; PC, phloem parenchyma cells.*

**FIGURE 7:**
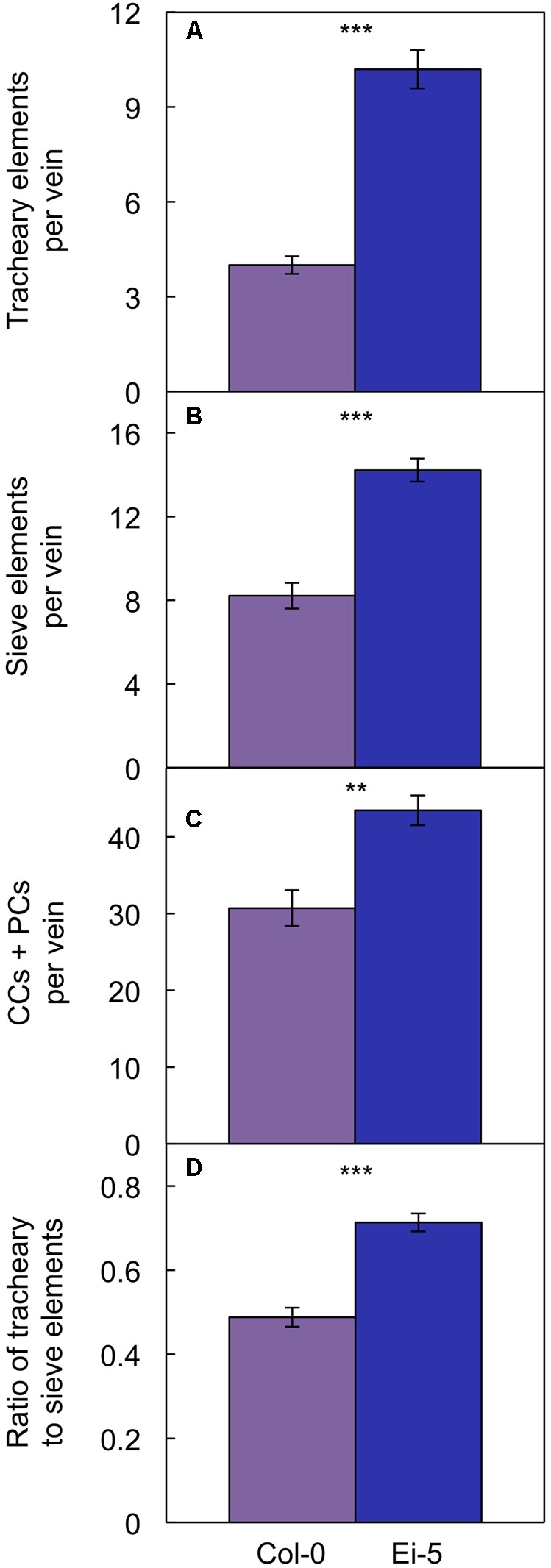
The number of **(A)** tracheary elements per minor vein, **(B)** sieve elements per minor vein, **(C)** companion cells (CCs) plus phloem parenchyma cells (PCs) per minor vein, and **(D)** ratio of tracheary to sieve elements in minor veins of the Col-0 (violet columns) and low-vein-density Ei-5 (indigo columns) accessions of *A. thaliana* grown under a 9-h photoperiod of 1000 μmol photons m^-2^ s^-1^ at a leaf temperature of 20°C. Mean values ± standard error, *n* = 4. The asterisks denote significant differences between the mean values; ^∗∗^*P* < 0.01, ^∗∗∗^*P* < 0.001.

Probing into the cell types comprising xylem or phloem confirmed these trends. Comparing Ei-5 with Col-0, the xylem’s tracheary elements were even more enhanced in their numbers per minor vein (**Figure 7A** and **Table 1**) than the phloem’s sieve elements (**Figure 7B** and **Table 1**) or companion cells and phloem parenchyma cells (**Figure 7C** and **Table 1**). These differential adjustments resulted in a higher ratio of water-transporting tracheary elements to sugar-transporting sieve elements in minor veins of Ei-5 compared to Col-0 that was, again, significant only under high growth light (**Figure 7D** and **Table 1**).

**FIGURE 8:**
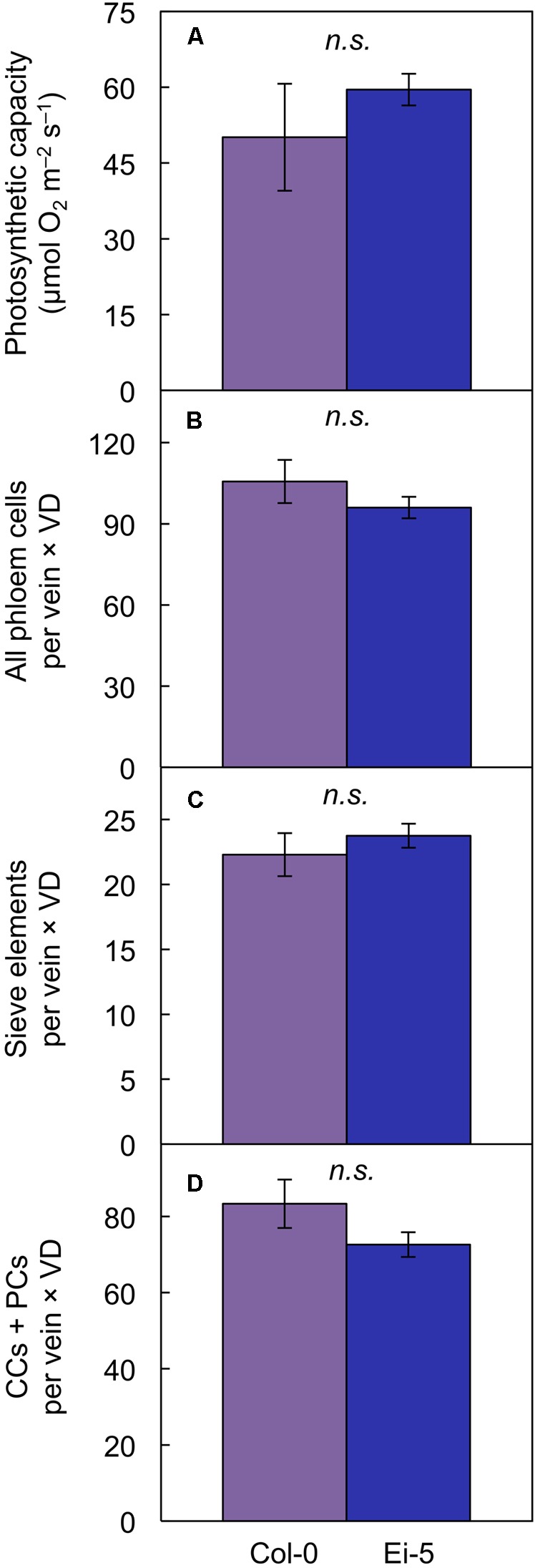
The **(A)** light- and CO_2_-saturated rate of photosynthetic oxygen evolution (photosynthetic capacity), **(B)** product of the number of phloem cells per minor vein and vein density, **(C)** product of the number of sieve elements per minor vein and vein density, and **(D)** product of the number of companion cells (CCs) plus phloem parenchyma cells (PCs) per minor vein and vein density in leaves of the Col-0 (violet columns) and low-vein-density Ei-5 (indigo columns) accessions of *A. thaliana* grown under a 9-h photoperiod of 1000 μmol photons m^-2^ s^-1^ at a leaf temperature of 20°C. Mean values ± standard deviations for **(A)** and ± standard errors for **(B–D)**; *n* = 4. There was no significant difference (*n.s.*) between any of the mean values.

Multiplying the cell number of various phloem cell types per minor vein × vein density revealed that there was no significant difference between the two accessions in phloem cell numbers per minor vein so normalized for vein density for leaves grown under high light (**Figures 8B–D**). This finding of no significant difference in cell number per minor vein when normalized for vein density applied to the sum of all phloem cells (**Figure 8B** and **Table 1**) as well as to those cells (sieve elements) serving as conduits for moving sugars out of the leaf (**Figure 8C** and **Table 1**) of leaves grown under either high or low light. For the cells involved solely in phloem loading (CCs + PCs), there was likewise no difference when normalized for vein density in leaves grown in high light (**Figure 8D**); however, there were slightly fewer phloem-loading cells in Ei-5 leaves grown in low light compared to Col-0 (**Table 1**). The similarity between the accessions in the number of phloem cells per minor vein after normalization for vein density is consistent with their similar photosynthetic capacity (**Figure 8A** and **Table 1**) and CO_2_ uptake for leaves grown in high light (29.4 ± 5.9 μmol m^-2^ s^-1^ vs. 32.7 ± 5.9 μmol m^-2^ s^-1^ in Col-0 vs. Ei-5, respectively).

**FIGURE 9:**
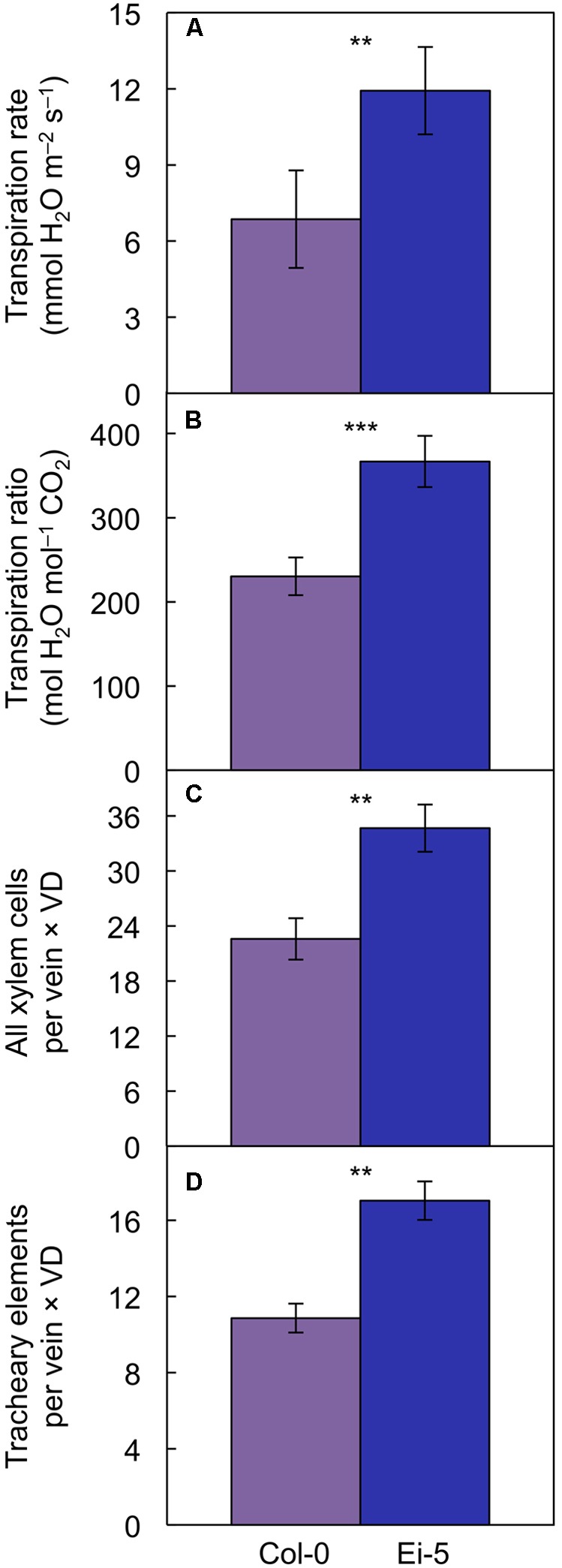
The **(A)** rate of transpirational water loss, **(B)** ratio of transpirational water loss to CO_2_ uptake, **(C)** product of the number of xylem cells per minor vein and vein density, and **(D)** product of the number of tracheary elements per minor vein and vein density in leaves of the Col-0 (violet columns) and low-vein-density Ei-5 (indigo columns) accessions of *A. thaliana* grown under a 9-h photoperiod of 1000 μmol photons m^-2^ s^-1^ at a leaf temperature of 20°C. Mean values ± standard deviations for **(A,B)** and ± standard errors for **(C,D)**; *n* = 4. The asterisks denote significant differences between the mean values; ^∗∗^*P* < 0.01, ^∗∗∗^*P* < 0.001.

However, transpiration rate per leaf area (**Figure 9A**) and the ratio of transpirational water loss to CO_2_ uptake (**Figure 9B** and **Table 1**) were both significantly higher in Ei-5 compared to Col-0, which is consistent with the difference in the ratio of tracheary elements to sieve elements between the two accessions (**Figure 7D** and **Table 1**). To make a more direct comparison to transpiration rate on a leaf area basis, the number of minor-vein xylem cells and tracheary elements was normalized for leaf area by multiplication × vein density, revealing significantly higher normalized numbers of both minor-vein xylem cells (**Figure 9C** and **Table 1**) and water-transporting tracheary elements (**Figure 9D** and **Table 1**) in Ei-5 compared to Col-0.

A two-way analysis of variance revealed a highly significant effect of genotype on all minor-vein and gas-exchange parameters with the exception of vascular cell numbers per minor vein normalized for vein density (**Table 2**). The impact of growth light intensity was also significant for all parameters except for the ratio of tracheary to sieve elements in the minor veins (**Table 2**). The interaction of genotype and growth light intensity was only significant for the number of vascular cells (xylem cells and tracheary and sieve elements) per minor vein and the transpiration ratio (**Table 2**).

**Table 2:** Results of a two-way analysis of variance for the effect of genotype, growth light intensity, and the degree of genotype response to growth light intensity for parameters in **Table 1** and scores of principal components 1 and 2 (see **Figure 10**).

Parameter	Genotype	Light	Genotype × Light
Minor vein density (mm mm^-2^)	^∗∗∗^	^∗∗^	*n.s.*
Cells per minor vein	^∗∗∗^	^∗∗∗^	^∗^
Cells per minor vein × vein density	*n.s.*	^∗∗∗^	*n.s.*
Xylem cells per minor vein	^∗∗∗^	^∗∗∗^	^∗∗^
Phloem cells per minor vein	^∗∗∗^	^∗∗∗^	*n.s.*
Ratio of xylem to phloem cells	^∗∗∗^	^∗^	*n.s.*
Tracheary elements per minor vein	^∗∗∗^	^∗∗∗^	^∗∗^
Sieve elements per minor vein	^∗∗∗^	^∗∗∗^	^∗^
CCs + PCs per minor vein	^∗∗∗^	^∗∗∗^	*n.s.*
Ratio of tracheary to sieve elements	^∗∗^	*n.s.*	*n.s.*
Photosynthetic capacity (μmol O_2_ m^-2^ s^-1^)	*n.s.*	^∗∗∗^	*n.s.*
Phloem cells per minor vein × vein density	*n.s.*	^∗∗∗^	*n.s.*
Sieve elements per minor vein × vein density	*n.s.*	^∗∗∗^	*n.s.*
CCs + PCs per minor vein × vein density	*n.s.*	^∗∗∗^	*n.s.*
Transpiration ratio (mol H_2_O mol^-1^ CO_2_)	^∗∗∗^	^∗∗∗^	^∗^
Xylem cells per minor vein × vein density	^∗∗^	^∗∗∗^	*n.s.*
Tracheary elements per minor vein × vein density	^∗∗∗^	^∗∗∗^	*n.s.*
Principal component 1	^∗∗∗^	^∗∗∗^	*^∗∗∗^*
Principal component 2	^∗∗∗^	^∗∗∗^	*^∗∗∗^*

*The asterisks denote significant effects; ^∗^P < 0.05, ^∗∗^P < 0.01, ^∗∗∗^P < 0.001, and n.s., not significant. CC, companion cells; PC, phloem parenchyma cells.*

**FIGURE 10:**
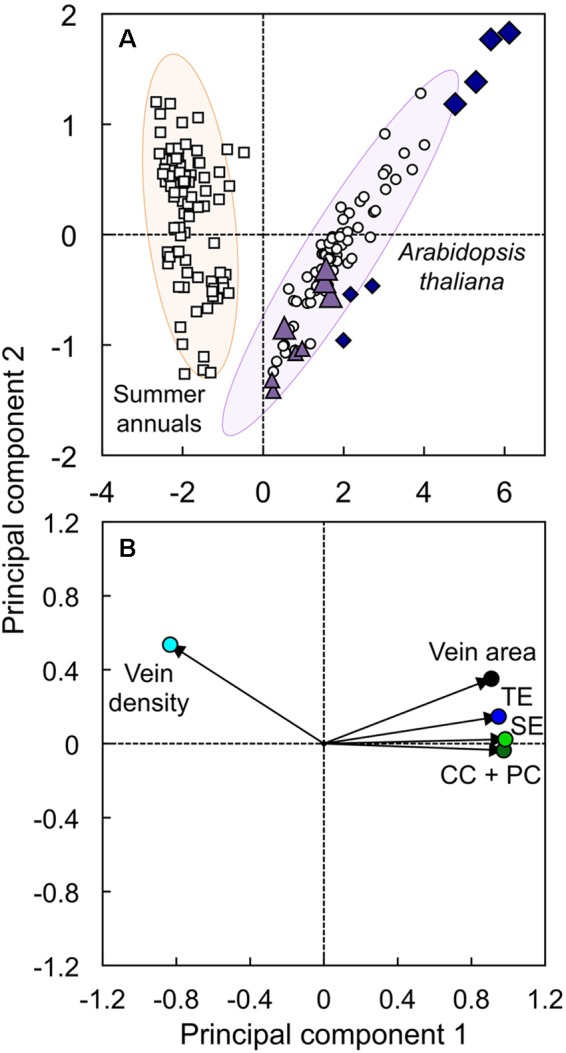
The **(A)** score and **(B)** loading plots from a principal component analysis of foliar vein density (light blue circle), minor vein cross-sectional area (black circle), and number of tracheary elements (TE; dark blue circle), sieve elements (SE; green circle), and companion cells plus phloem parenchyma cells (CC + PC; dark green circle) per minor vein in leaves of the Col-0 (violet triangles) and low-vein-density Ei-5 (indigo diamonds) accessions of *A. thaliana* grown under a 9-h photoperiod of 100 (small triangles and diamonds) or 1000 (large triangles and diamonds) μmol photons m^-2^ s^-1^ at a leaf temperature of 20°C, as well as leaves of Col-0, Italian and Swedish ecotypes of *A. thaliana* (open circles; *n* = 67), and summer annual crop species (open squares; *n* = 83) grown under multiple experimental conditions. Principal component 1 explained 86.7% of the variation in the data, and principal component 2 explained 8.64% of the variation in the data. Density ellipses (*P* = 0.950) of the summer-annual (orange ellipse) and *A. thaliana* (purple ellipse) groups have been superimposed behind the score plot. Data for *A. thaliana* from Cohu et al. (2013a,b), Adams et al. (2016), and Stewart et al. (2017a,b), and data for summer annuals from Cohu et al. (2014); Muller et al. (2014b), and Polutchko et al. (2018)

Principal component analysis was conducted to evaluate the difference in overall vascular organization between the low-vein-density Ei-5 accession and various other *A. thaliana* accessions and several summer annual crops (**Figure 10**). This analysis included minor-vein density and cross-sectional area, as well as the number of sieve elements, tracheary elements, and the sum of companion cells plus phloem parenchyma cells per minor vein in leaves of Ei-5, Col-0, two *A. thaliana* ecotypes from Sweden and Italy (Ågren and Schemske, 2012; see also Adams et al., 2016), and eight summer annual crops: cotton, cucumber, pumpkin, squash, sunflower, tobacco, tomato, and watermelon (for more details, see Muller et al., 2014b; Polutchko et al., 2018). The first principal component (PC1) explained 86.7% of the variation in this data set and was driven negatively by minor-vein density and positively by minor-vein cross-sectional area and cell numbers (**Figure 10B**). The summer annuals and *A. thaliana* completely separated along PC1, with negative PC1 scores for all summer annuals and positive PC1 scores for all *A. thaliana* (**Figure 10A**). Density ellipses (*P* = 0.950) encapsulated 80 out of 83 summer annuals and 73 out of 78 *A. thaliana* points, with five of seven Ei-5 points falling outside of the latter ellipse (**Figure 10A**). In addition, all four Ei-5 points from the high light plants had more positive PC1 scores than all of the other 74 *A. thaliana* points, and the three Ei-5 points from low light had PC1 scores that were considerably more positive than their low light Col-0 counterparts (**Figure 10A**). The second principal component was driven primarily by foliar vein density and vein cross-sectional area, although it should be noted that this component explained less than 10% of the variation in the data set. The effects of genotype and growth light intensity, as well as the interaction of genotype and growth light intensity, were highly significant for the scores of the first two principal components (**Table 2**). In summary, the accessions of the winter annual *A. thaliana* formed a cluster that segregated completely from summer annual species, with lower vein density, larger veins, and greater numbers of all vascular cell types in the winter annual. Ei-5 aligned with all of the other *A. thaliana* ecotypes, but extended the range to even lower vein densities and larger veins with greater numbers of vascular cells.

## Discussion

The findings of this study add to a body of literature (Adams et al., 2013a, 2014a, 2016; Cohu et al., 2013b, 2014; Muller et al., 2014a,b; Stewart et al., 2016, 2017a,b, 2018; Polutchko et al., 2018) demonstrating a strong relationship between photosynthetic capacity and several minor-vein parameters; these parameters include proxies for the capacity for sugar loading into foliar phloem and export out of the leaf as well as parameters associated with water import into the leaf and transpirational water loss relative to photosynthesis. The present findings provide further evidence of compensatory adjustments in minor-vein architecture that contribute to the adaptation and acclimation of photosynthetic capacity. Adjustments seen in Ei-5 apparently facilitate a level of sugar loading and export from the leaf mesophyll tissue equivalent to that of Col-0 with its higher foliar vein density. It is likely that maintenance of sugar-export capacity in Ei-5 despite its low vein density prevents sugar build-up in the leaves and thereby permits maintenance of photosynthetic capacity in Ei-5 (see Ainsworth and Bush, 2011; Adams et al., 2013a; Nikinmaa et al., 2013). The importance of a high sink strength comprised of the plant’s growing, reproductive, and/or storage tissues in keeping up the demand for photosynthate has been well documented (Körner, 2013; Fatichi et al., 2014). Avoiding limitations to photosynthetic performance by insufficient sugar export from leaves can be thought of as removing yet another “sink limitation.” Specifically, the present study demonstrates that a greater number of phloem cells per minor vein fully compensates for the genetic constraint of low vein density in the Ei-5 accession.

While the low-vein-density accession Ei-5 exhibited compensatory generation of greater numbers of phloem cells in each of its fewer foliar minor veins, the xylem tissue showed over-proportional adjustment in the number of xylem cells. This xylem adjustment in Ei-5 apparently provided for enhanced hydraulic capacity, thus supporting a significantly higher rate of transpiration compared to Col-0, which is a novel and somewhat unexpected combination of features in view of the findings from previous studies. In all previously documented cases of leaves with a greater number of tracheary elements per minor vein and higher ratios of tracheary to sieve elements, such leaves also had greater vein densities (Adams et al., 2016; Stewart et al., 2016, 2017a, 2018). It is unknown whether the combination of reduced foliar vein density and enhanced foliar water-flux capacity of Ei-5 compared to Col-0 is maladaptive or has any ecological significance. One may speculate that the geographic Ei-5 location, with its relatively high precipitation level (**Figures 1E,F**), may offer more support to the low-vein density phenotype of Ei-5 than a location with lower precipitation levels. Comparison of vein density among several *A. thaliana* ecotypes from locations with different precipitation levels revealed that the ecotype from the driest location had the highest vein density (Adams et al., 2016). In support of the possibility that the relatively high and consistent precipitation experienced by Ei-5 in its native habitat permits persistence of this phenotype, the Ba-1 accession, noted by Candela et al. (1999) as the only other *A. thaliana* accession in their survey with an exceptionally low vein density (see also Pérez-Pérez et al., 2009), originates from a site near Blackmount, United Kingdom (56.5°N, 4.8°W) that receives 1824 mm in annual precipitation – more than threefold greater than the site from which Col-0 originates.

Another possible explanation for its the unusual vascular composition is that Ei-5 may compensate for limitations in water flux that could be inherent to having only very few, large veins by preferential augmentation of minor-vein xylem tissue (see Sack and Scoffoni, 2013). A greater number of xylem cells per minor vein of Ei-5 presumably not only increases water-flux capacity through tracheary element conduits but also provides a greater surface area at the interface between xylem and adjacent mesophyll tissues for enhanced water distribution across the lamina compared to the scenario with fewer xylem cells per vein in the Col-0 accession (for discussions of these issues among species with different vein densities, see Prado and Maurel, 2013; Buckley et al., 2015).

Photosynthetic capacity per leaf area was similar in Ei-5 and Col-0 despite a significantly lower vein density, altered minor vein architecture, and significantly reduced shoot growth in Ei-5. These reduced growth rates of Ei-5, and its smaller rosettes compared to Col-0, are curious given the ability of Ei-5 to maintain similar photosynthetic capacities per leaf area. This finding suggests that there may be a link between reduced rosette development and the auxin-signaling-related *hve-1* allele that gives rise to the low-vein-density phenotype in Ei-5 (Alonso-Peral et al., 2006). Coordination of venation and leaf growth via genetic control was also suggested to explain reduced vein densities and leaf sizes of *hve* mutants in the Col-0 background (Pérez-Pérez et al., 2011; see also Dengler and Kang, 2001). Another explanation for reduced growth in Ei-5 compared to Col-0 is a possible impact of low vein density on the growth-driving water potential of the mesophyll cells furthest from the nearest minor vein (see discussion in Buckley et al., 2015). Such genetic and biophysical effects may also offer an explanation for relatively high growth rates of many summer annual crops with relatively high vein densities compared to *A. thaliana* despite similar photosynthetic capacities and sugar-exporting tissues per leaf area (see Demmig-Adams et al., 2014; Polutchko et al., 2018).

## Author Contributions

WA and BD-A designed the experiments. JS and SP carried out the experiments and collected the data. JS analyzed the data, assembled the climatological data, executed the statistical analyses, and rendered the figures. WA, JS, and BD-A wrote the manuscript.

## Conflict of Interest Statement

The authors declare that the research was conducted in the absence of any commercial or financial relationships that could be construed as a potential conflict of interest.

## Footnotes

1 https://www.arabidopsis.org

2 http://1001genomes.org/tables/master_accession_list.html

3 http://www.nhc.noaa.gov/gccalc.shtml

4 http://aa.usno.navy.mil/data/docs/Dur_OneYear.php

5 http://www.worldclim.org

6 http://www.diva-gis.org
